# Experimental Study on RAP with High Recycling Content Based on High-Modulus Asphalt Mixture

**DOI:** 10.3390/ma18122835

**Published:** 2025-06-16

**Authors:** Xin Wang, Bangwei Wu, Zhengguang Wu, Bo Li

**Affiliations:** 1 College of Civil Science and Engineering, Yangzhou University, Yangzhou 225127, China; wangxin_yzu@126.com (X.W.); zgwu@yzu.edu.cn (Z.W.); liboyzu@163.com (B.L.); 2 Research Center for Basalt Fiber Composite Construction Materials, Yangzhou University, Yangzhou 225127, China

**Keywords:** reclaimed asphalt pavement, hard asphalt, pavement performance, dynamic modulus

## Abstract

To improve the recycling content of Reclaimed Asphalt Pavement (RAP), this paper utilizes the characteristic of aged and hardened asphalt in RAP materials by adopting the High-modulus Asphalt Mixture design method for high-RAP-content recycling. First, the basic technical performance, fatigue properties, rheological characteristics, and chemical functional groups of reclaimed asphalt, 30# hard asphalt, and Styrene-Butadiene-Styrene (SBS)-modified asphalt were analyzed. The results revealed significant similarities in various metrics between reclaimed and hard asphalt, demonstrating the feasibility of replacing hard asphalt with reclaimed asphalt in a High-modulus Asphalt Mixture design. Next, High-modulus Asphalt Mixtures, EME13, with different RAP contents (0%, 20%, 40%, 60%) were designed and compared with SBS-modified Sup13 mixtures. The results indicated that (1) as the RAP content increased, the high-temperature performance of EME13 improved by 20~60%, while its low-temperature and intermediate-temperature crack resistance slightly declined by 10~20%. The dynamic modulus in the low-frequency region increased by 3~6 times, whereas the high-frequency dynamic modulus decreased by 20~30%. RAP enabled EME13 to meet the modulus design requirements more readily for High-modulus Asphalt Mixtures. (2) Although the SBS-modified Sup13 exhibited superior pavement performance compared to EME13, its cost was significantly higher. EME13 with high RAP content demonstrated notable economic advantages despite slightly lower pavement performance than Sup13. This research provides a new technical approach for the high-content recycling of RAP materials.

## 1. Introduction

Recycling asphalt pavement materials can conserve resources, reduce engineering costs, and promote environmental protection [1]. With the continuous development of plant-mixed hot recycling technology, the pavement performance of recycled asphalt mixtures has approached that of virgin asphalt materials under conditions of RAP content under a specific limit. Ma’s [2] research findings indicate that with the increase in RAP content, the high-temperature performance of AC13 improves, while the water stability and anti-cracking performance first increase and then decrease. It is recommended that the suitable RAP content be 20~40%. Kaseer [3] investigated the field performance of reclaimed asphalt pavement and reached similar conclusions based on the investigation findings. However, considering RAP utilization’s significant economic and environmental benefits, Li [4] pointed out that how to optimize the RAP content increment remains a critical challenge. Some efforts have been made on this aspect. Warm-mix technology is considered a feasible approach to increase RAP content. Li [5] studied the rheological properties of warm-mix recycled asphalt. The results show that when the content of recycled asphalt reaches up to 50%, the warm-mix recycled asphalt still exhibits good high-temperature deformation resistance and low-temperature crack resistance. Pei [6] developed a new type of asphalt rejuvenator suitable for warm-mix recycled asphalt mixtures with high RAP content using molecular simulation methods. However, some studies indicate that warm-mix additives have some shortcomings. Liu [7] adopted foamed warm asphalt in PAP mixtures. The results showed that warm-mix formulations had improved low-temperature performance. However, their high-temperature stability was poorer, and water stability was lower. Further, using the molecular simulation method, Jin [8] investigated the interfacial molecular motion of warm additives, asphalt, and aggregates from a thermodynamic perspective. The results revealed the micro-mechanism by which warm additives weaken the water stability of asphalt mixtures. Therefore, some scholars are exploring other methods to increase RAP content. For example, Islam [9] used waste engine oil and waste cooking oil as a rejuvenator, and Karimi [10] adopted nano calcium carbonate as a modifier to enhance the cracking resistance of warm mix asphalt mixtures prepared with high contents of RAP. Besides, an advanced mixture design method, i.e., the French High-modulus Asphalt Mixture design method, shows unique advantages.

The High-modulus Asphalt Mixture technology originated in France in the 1980s, and the French Center for Road and Bridge has systematically improved its design method since 2004. Compared with traditional dense-graded asphalt concrete, Wielinski [11] summarized that this technology has four key characteristics: (1) the use of hard asphalt with penetration about 20 or high-modulus modified asphalt, (2) the adoption of finer gradation with significantly increased filler content, (3) the asphalt-aggregate ratio elevated to 5.0–7.0%, and (4) the compacted air voids reduced to 1.5–3.0%. Its technical principle ensures high-temperature stability through hard asphalt while maintaining fatigue resistance via high asphalt content. Notably, the aged asphalt in RAP shares similar hardening characteristics with hard asphalt. Bilema [12] argued that the penetration of aged SBS asphalt is about 30, equivalent to 30# hard asphalt. Li [13] compared the technical properties of four types of SBS asphalt after ageing. The results showed that although the rheological properties of the different SBS asphalts after ageing were significantly different, their penetration values were quite close, equivalent to 20# hard asphalt. Inspired by this phenomenon, researchers propose replacing new asphalt with aged asphalt in RAP. Zaumanis [14] validated this concept’s feasibility. He found that although a 100% RAP asphalt mixture cannot possibly fully fulfill the fatigue, modulus, and rutting requirements of High-modulus Asphalt Mixtures, the design method for High-modulus Asphalt Mixtures can significantly increase the RAP content. Jian [15] used Trinidad Lake asphalt and RAP to prepare a High-modulus Asphalt Mixture. The test results showed that the performance of the asphalt mixture with 50% RAP was similar to that of AC-20, demonstrating that recycling aged asphalt using hard asphalt binder for hot-mixing recycled asphalt mixture to increase the RAP content is feasible. Although this breakthrough shows significant application potential, it is still necessary to verify the applicability of RAP from different sources and to deeply evaluate the comprehensive technical properties of high-RAP-content recycled mixtures.

Therefore, based on the French EME-13 gradation standard, this study designed high-modulus recycled mixtures with four RAP contents of 0%, 20%, 40%, and 60%, and the conventional SBS-modified asphalt mixture Sup-13 was taken as the control group. After analyzing the technical performance similarities of reclaimed asphalt, 30# hard asphalt, and SBS-modified asphalt, the dynamic modulus, high-temperature stability, cracking resistance, and cost-effectiveness of the mixtures are systematically analyzed to provide a new technical approach for high-content RAP recycling technology.

## 2. Materials

### 2.1. Asphalt

The virgin asphalt used in this study included 30# pure asphalt and SBS-modified asphalt. The former was employed to prepare EME13 High-modulus Asphalt Mixtures, while the latter was used to fabricate Sup13-modified asphalt mixtures as the control group. Additionally, aged asphalt was recovered from RAP materials for comparative analysis to evaluate performance differences between recycled and virgin asphalt.

### 2.2. Aggregates and Filler

The virgin aggregates used in this study include basalt and limestone, with aggregates exceeding 3 mm in particle size being basalt and those below 3 mm being limestone. The RAP material was sourced from a national highway in Jiangsu Province and preprocessed at an asphalt mixing plant. The filler employed was limestone mineral powder. Sieve analysis results for all aggregates are presented in Table 1.

## 3. Methods

### 3.1. Asphalt Performance Testing Methods

#### 3.1.1. Basic Properties Testing Methods

The basic properties of asphalt include penetration, ductility, and softening point. These were tested according to the methods T0604, T0605, and T0606 in the Standard Test Methods of Bitumen and Bituminous Mixtures for Highway Engineering (JTG E20-2011) [16].

#### 3.1.2. Linear Amplitude Sweep

Linear Amplitude Sweep (LAS) was conducted according to the standard method specified in AASHTO TP 101-18 [17] to evaluate the fatigue performance of asphalt binders. The test was performed using a dynamic shear rheometer (DSR) from TA Instruments at a temperature of 25 °C. The asphalt specimen was subjected to strain scanning at a constant frequency of 10 Hz, with the strain increasing from 0.1% to 30% over 3100 loading cycles. The data obtained during the test were analyzed using the viscoelastic continuous damage theory, ultimately deriving the asphalt fatigue equation, as shown in Equation (1).

(1) $$N_{f}=A{\left(\gamma_{\max}\right)}^{-B}$$

where *N_f_* is the fatigue life of asphalt binders, *A* and *B* are the fatigue parameters, and *γ_max_* is the strain.

It should be noted that the software directly obtained Equation (1) through automatic analysis of test data, and no additional work was required from experimenters. Considering that fatigue life test results usually have high variability, the research conducted eight sets of tests for each type of asphalt.

#### 3.1.3. Bending Beam Rheometer (BBR) Test

The BBR test was conducted using AASHTO T 313-22 [18]. This test was used to evaluate the cracking resistance of asphalt binders at low temperatures, with testing performed at −12 °C, −18 °C, and −24 °C, respectively. A bending beam specimen of asphalt (dimensions: 127 mm × 12.7 mm × 6.35 mm) was subjected to a load of 980 mN ± 50 mN for a duration of 240 s. The creep stiffness (*S*) and creep rate (*m*) of the asphalt specimen were measured during this process.

#### 3.1.4. Fourier-Transform Infrared Spectroscopy (FTIR) Scan

FTIR was used to analyze the chemical functional groups of different asphalts. This employed the wavenumber range of 4000–400 cm^−1^. The chemical composition characteristics of various asphalts were examined on the FTIR spectral features. This analysis provided insights into molecular structural differences and ageing-related chemical changes in asphalt.

### 3.2. Asphalt Mixture Performance Tests

#### 3.2.1. Wheel Tracking Test

This test evaluated the rutting resistance of asphalt mixtures. The procedure followed the T0719 test method specified in the Standard Test Methods of Bitumen and Bituminous Mixtures for Highway Engineering (JTG E20-2011) [17]. The specimen size was a rectangular prism measuring 300 mm × 300 mm × 50 mm, and the test temperature was 60 °C. The test result is expressed as dynamic stability (DS), and its calculation formula is given in Equation (2).

(2) $$DS=\frac{(t_{2}-t_{1})\times N}{d_{2}-d_{1}}\times C_{1}\times C_{2}$$

where *DS* is the dynamic stability of the asphalt mixture (passes/mm); *t*_1_ and *t*_2_ are the loading times at 45 min and 60 min, respectively; *d*_1_ and *d*_2_ are the deformation depths at *t*_1_ and *t*_2_ (mm); and *C*_1_ and *C*_2_ are the correction factors, which are usually 1.0. *N* is the load frequency of the rubber wheel, equal to 42 passes/min.

#### 3.2.2. Low-Temperature Bending Beam Test

**Figure 1:**
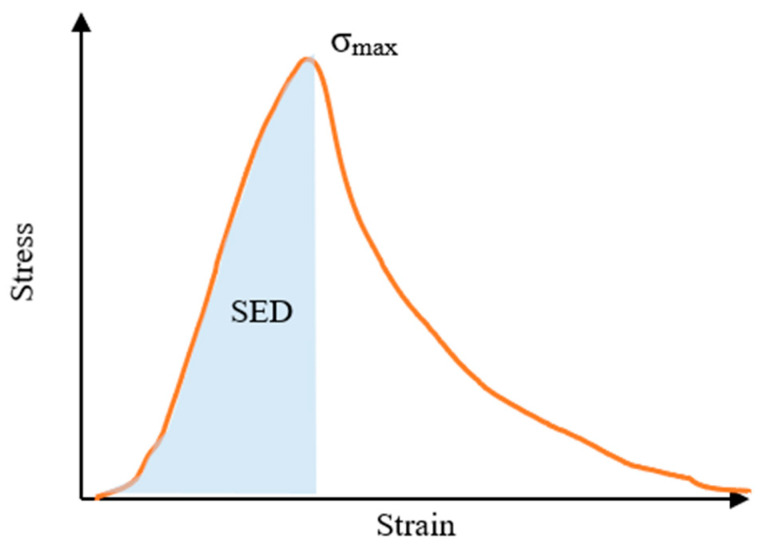
Schematic diagram of SED.

This test was used to evaluate the low-temperature cracking resistance of asphalt mixtures. The procedure followed the T0715 test method specified in the Standard Test Methods of Bitumen and Bituminous Mixtures for Highway Engineering (JTG E20-2011) [17]. The test specimen was a rectangular prism measuring 250 mm × 30 mm × 35 mm, and it was subjected to three-point bending loading at a loading rate of 50 mm/min. The test temperature was −15 °C. Two indicators were used to assess the low-temperature cracking resistance of the asphalt mixture. One was the strain at specimen failure, and the other was strain energy density (SED). Many researchers believed that SED is closely related to the low-temperature performance of asphalt mixtures [19]. SED refers to the area enclosed by the stress–strain relationship curve, as shown in Figure 1.

#### 3.2.3. IDEAL Cracking Test (IDEAL-CT)

IDEAL-CT was a testing method used to determine the cracking resistance of asphalt mixtures [19]. The procedure was the same as the conventional tensile splitting test. At 25 °C, a cylindrical specimen with a diameter of 100 mm and a height of 63 mm was subjected to a splitting test at a loading rate of 50 mm/min. The CT_index_ is calculated based on the test results, as shown in Equation (3).

(3) $$CT_{index}=\frac{G_{f}}{\left|m_{75}\right|}\times \frac{l_{75}}{D}$$

where *G_f_* is the fracture energy (J/m^2^), calculated by dividing the work of failure (area under the load-displacement curve) by the cross-sectional area of the specimen; |*m*_75_| is the absolute value of the slope of the load–displacement curve at the point where the load is reduced to 75% of the peak load; *l*_75_ is the displacement at the point where the load is reduced to 75% of the peak load (mm); and *D* is the specimen diameter (mm).

#### 3.2.4. Dynamic Modulus Test

This test was conducted by AASHTO T 342-22 [20]. The test involved applying continuous sinusoidal axial loads to the specimen to obtain its stress–strain response, from which the dynamic modulus of the specimen was calculated. The test specimen size was a cylindrical shape with a diameter of 100 mm and a height of 150 mm. The test selected five temperatures (−10 °C, 5 °C, 20 °C, 35 °C, and 50 °C) and five frequencies (0.1 Hz, 1 Hz, 5 Hz, 10 Hz, and 20 Hz), and the dynamic modulus master curve of the asphalt mixture was fitted using the sigmoidal function.

## 4. Results and Discussions

### 4.1. Test Results of Asphalt Properties

#### 4.1.1. Basic Properties Test Results

The test results of the softening point, penetration, and ductility of different asphalts are shown in Table 2.

The RAP material used in this study was initially constructed with SBS-modified asphalt. The test results in the table indicate that the SBS-modified asphalt exhibited ageing after years of service in the pavement. Compared to the original SBS-modified asphalt, the penetration, softening point, and ductility of the reclaimed asphalt decreased by 49.2%, 19.3%, and 89.5%, respectively. This degradation is due to the decomposition of the SBS modifier under traffic loading and environmental effects, as well as the volatilization of the lighter components in the asphalt, which causes the hardening of the asphalt [21,22]. Moreover, the basic properties of reclaimed asphalt are quite close to those of 30# hard asphalt. Regarding penetration, reclaimed asphalt and 30# hard asphalt are the same grade. This indicates that it is feasible to use hardened reclaimed asphalt as a substitute for hard asphalt in High-modulus Asphalt Mixtures.

#### 4.1.2. LAS Test Results

The fatigue equations for different asphalts were fitted based on the LAS test results, and the fatigue life of the asphalts under different strain levels was calculated. The results are shown in Table 3.

The results indicate that SBS-modified asphalt exhibits the highest fatigue life. For example, at a strain level of 2.5 ε, the fatigue life of SBS asphalt is 2.3 times and 3.5 times that of reclaimed and hard asphalt, respectively. This is because the SBS modifier has improved the tensile properties of the asphalt, making it less prone to fatigue under repeated stretching [23].

Further comparison of the fatigue life of SBS asphalt and reclaimed asphalt shows that the parameter B of reclaimed asphalt increases after ageing, indicating that the fatigue life of reclaimed asphalt is more sensitive to strain levels. In addition, the fatigue life of the reclaimed asphalt used in this study is higher than that of hard asphalt, with a fatigue life of 1.5 to 1.6 times that of hard asphalt. Moreover, the parameter B of reclaimed asphalt is also lower. This may be due to the incompletely undegraded SBS modifier in the reclaimed asphalt that remains effective.

#### 4.1.3. BBR Test Results

**Figure 2:**
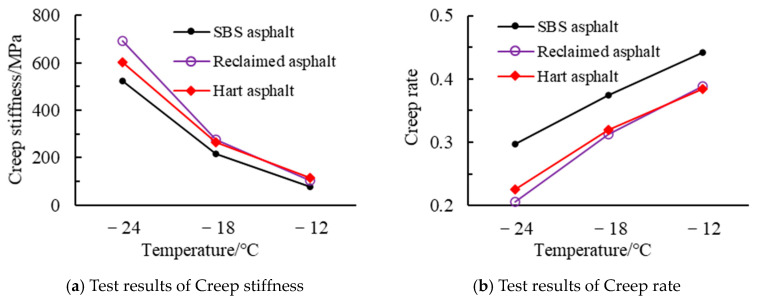
BBR test results of different asphalts.

The BBR test results for different asphalts are shown in Figure 2. A higher creep stiffness indicates a harder asphalt, while a higher creep rate reflects stronger stress relaxation capability. As shown in Figure 2, with the decrease in temperature, the creep stiffness of the three asphalts increases and the creep rate decreases, indicating that lower temperatures lead to poorer crack resistance in asphalt. Furthermore, SBS-modified asphalt consistently exhibits the best cracking resistance across all tested temperatures. Its creep rate is approximately 15–30% higher than that of other asphalts, which is attributed to the SBS modifier enhancing the asphalt’s flexibility, thereby making it less likely to fracture at low temperatures due to deformation.

Furthermore, it can be observed from Figure 2 that at −24 °C, the creep stiffness of reclaimed asphalt and hard asphalt differs by about 13%, and the creep rate differs by about 9%. At −18 °C, the differences are approximately 10% and 1%, respectively. This indicates that the low-temperature properties of reclaimed asphalt and hard asphalt are quite similar, further proving the feasibility of using reclaimed asphalt as a substitute for hard asphalt in High-modulus Asphalt Mixtures.

#### 4.1.4. FTIR Test Results

**Figure 3:**
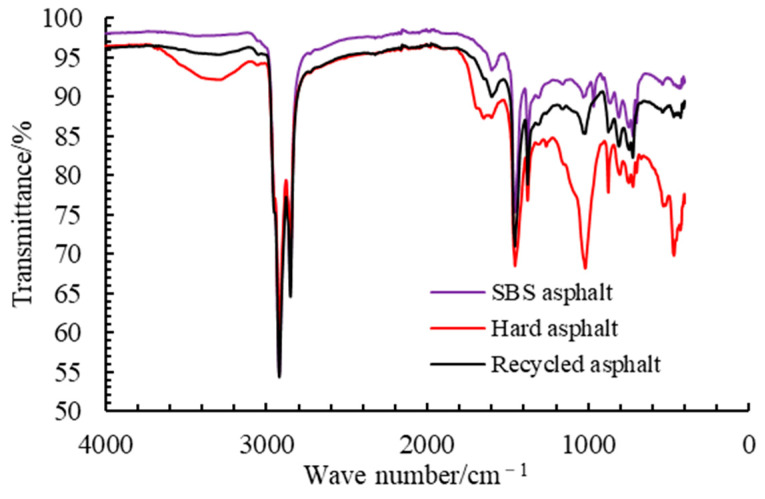
FTIR test results of different asphalts.

**Figure 4:**
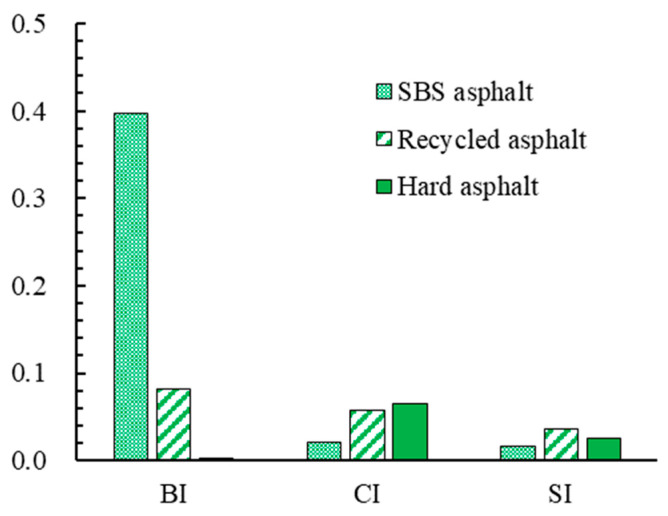
Functional group index calculation results.

The FTIR test results are shown in Figure 3. To analyze the infrared spectral characteristics of different asphalts, this paper calculates the carbonyl index (CI), sulfoxide index (SI), and butadiene index (BI) of each asphalt based on the changes in the carbonyl absorption peak at 1700 cm^−1^, the sulfoxide absorption peak at 1030 cm^−1^, and the butadiene absorption peak at 966 cm^−1^. These three functional group indices can characterize the distribution of functional groups in asphalt from different perspectives [24,25]. The calculation formulas are given in Equation (4), and the results are shown in Figure 4.

(4) $$I_{i}=A_{i}/\;\Sigma A$$

where *I_i_* represents the functional group index, *A_i_* represents the area of the functional group absorption peak, and *ΣA* represents the sum of the absorption peak areas within the wavenumber range of 4000 cm^−1^ to 400 cm^−1^.

As shown in Figure 4, (1) in terms of BI, SBS asphalt has the highest BI, followed by reclaimed asphalt, with hard asphalt having the lowest BI. BI is calculated based on the absorption peak at 966 cm^−1^, which is caused by the C=C bonds in the butadiene of the SBS modifier. Therefore, it can be seen that the degradation of the SBS modifier in reclaimed asphalt leads to a decrease in BI, while hard asphalt, which does not contain the SBS modifier, also has a lower BI. (2) Regarding CI and SI, it can be observed that the values of reclaimed asphalt and hard asphalt are both higher than those of SBS asphalt. The changes in CI and SI are due to the vibrations of the carbonyl group at 1700 cm^−1^ and the sulfoxide group at 1030 cm^−1^, respectively. During the ageing process of asphalt, C=C bonds or alkanes can be oxidized to form ketone/carboxylic acid compounds, leading to an increase in CI; while sulfur-containing compounds can be oxidized to form sulfoxide groups, resulting in an increase in SI. Reclaimed asphalt has undergone the ageing process and has higher CI and SI values. Although the hard asphalt used in this study has not experienced ageing, its CI and SI values are closer to those of reclaimed asphalt. This indicates that the chemical composition of reclaimed asphalt is closer to that of hard asphalt than SBS-modified asphalt. Therefore, using reclaimed asphalt instead of hard asphalt to prepare High-modulus Asphalt Mixtures is entirely feasible.

### 4.2. Test Results of Asphalt Mixture Performance

#### 4.2.1. Design of Asphalt Mixtures

In this study, four types of high-modulus asphalt mixtures with different RAP contents were designed, with RAP contents of 0%, 20%, 40%, and 60%, respectively, denoted as EME-0, EME-20, EME-40, and EME-60. Additionally, a Sup13 asphalt mixture was designed as a control group. The gradation curves of the five asphalt mixtures are summarized in Table 4, and their mix design results are presented in Table 5.

As seen from Table 4 and Table 5, the gradation of EME13 is finer than that of Sup13, as evidenced by its higher passing rate at 0.075 mm. Additionally, EME13 has a higher asphalt content and a lower designed air void. These distinct characteristics of the asphalt mixtures will affect their pavement performance.

#### 4.2.2. Wheel Tracking Test Results

**Figure 5:**
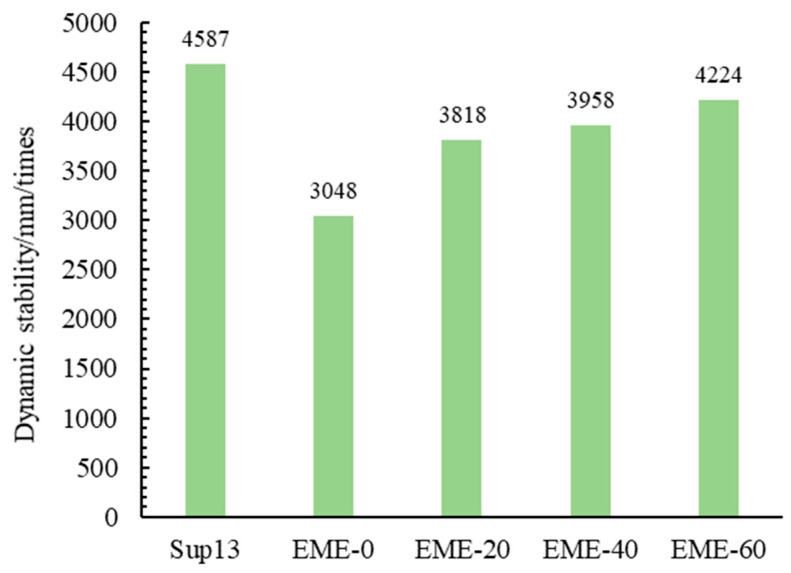
The dynamic stability of different asphalt mixtures.

The dynamic stability test results of different asphalt mixtures are shown in Figure 5.

As can be seen from the results, (1) as the RAP content in EME13 increases, its dynamic stability progressively rises. Compared to EME-0 (0% RAP), the dynamic stability of mixtures with 20%, 40%, and 60% RAP increases by 25.2%, 29.8%, and 38.6%, respectively. This is because the softening point of the reclaimed asphalt in RAP is higher than that of hard asphalt (as shown in Table 2), which improves high-temperature performance. Consequently, higher RAP content enhances resistance to rutting. (2) The dynamic stability of Sup-13 is 7.9% higher than that of EME-60, showing the best rutting resistance. This is because Sup-13 uses SBS-modified asphalt with a higher softening point, and its asphalt content is only 4.9%, lower than the 5.3% of EME-13. The lower asphalt content is also the reason for the higher dynamic stability of Sup-13.

#### 4.2.3. Low-Temperature Bending Beam Test Results

**Figure 6:**
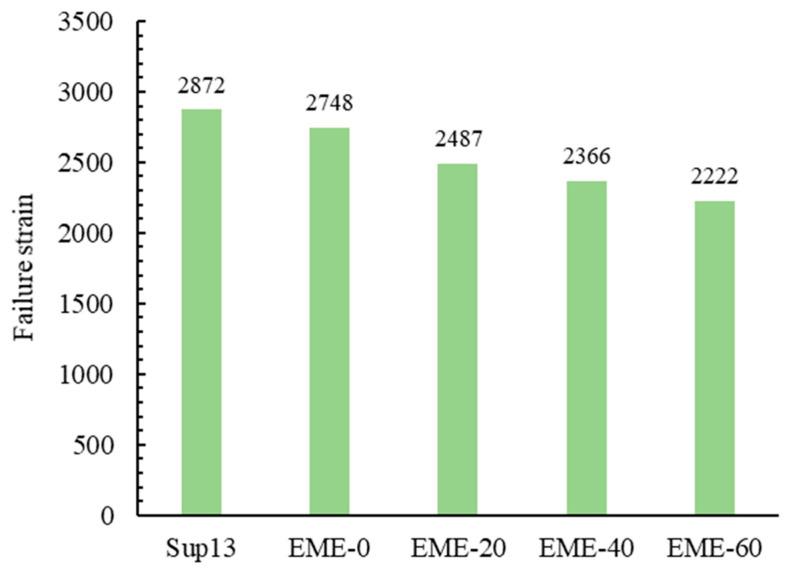
The failure strain of different asphalt mixtures.

**Figure 7:**
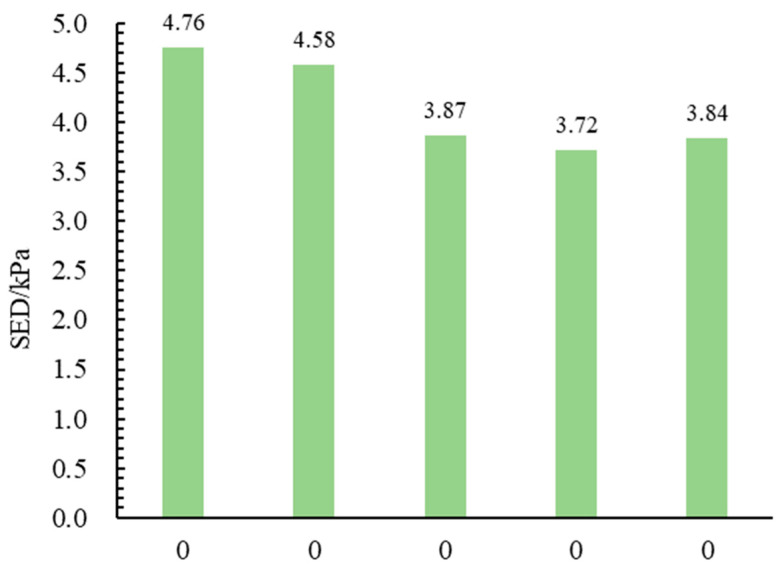
The SED of different asphalt mixtures.

Figure 6 and Figure 7 show the low-temperature bending beam test results. The two figures present the result of failure strain and SED, respectively. Each test result is averaged from four specimen test groups.

As shown in Figure 6, (1) the low-temperature failure strain of EME-13 decreases with the increase in RAP content. This is attributed to the reduced performance of reclaimed asphalt under low-temperature conditions. During the ageing process, asphalt undergoes oxidation, polymerization, and volatilization of light components, leading to increased stiffness and greater susceptibility to brittle fracture at low temperatures. Test results indicate that when RAP content varies between 20% and 60%, the reduction in failure strain ranges from 10% to 20%, suggesting a slight decline in the low-temperature crack resistance of EME-13. These findings are generally consistent with the BBR test results for asphalt binders. (2) When comparing EME-13 with Sup-13, it is found that as the RAP content increases from 0% to 60%, the low-temperature failure strain of EME-13 is lower than that of Sup-13, but the difference is within 20%. For instance, the failure strain of EME-0 is only 4.3% lower than that of Sup-13. This indicates that the low-temperature cracking resistance of Sup-13 is only slightly better than that of EME-13. Although Sup-13 uses SBS-modified asphalt with better low-temperature performance, the finer grading and higher asphalt content of EME-13 contribute to improving the low-temperature performance of the asphalt mixture. Consequently, EME-13 shows only a minor reduction in failure strain compared to Sup-13.

In asphalt pavements, the thermal stress generated in winter temperatures cannot be relaxed in time, and energy will continue to accumulate over time. Once it reaches the limit allowed by the asphalt mixture, it will be released from the weak parts of the pavement in the form of cracking. Therefore, many researchers believe that it is more reasonable to describe the low-temperature performance of asphalt mixture from the perspective of energy [26].

As shown in Figure 7, (1) with the RAP content increasing from 0% to 60%, the SED first decreases and then slightly increases, with a variation of less than 20%. The SED of EME-0 is 4.58 kPa, EME-40 drops to the lowest, 3.72 kPa, and EME-60 rises to 3.84 kPa. This indicates that moderately increasing the RAP content will reduce SED, while too high content will restore the strain energy density to a certain extent. As the RAP content increases, the asphalt mixture gradually hardens, its failure strain decreases, and its failure strength gradually increases. SED is a comprehensive reflection of the failure strain and failure strength. (2) The SED value of Sup13 is 4.76 kPa, which is significantly higher than that of EME13. This is because Sup13 uses SBS-modified asphalt. Compared with the 30# hard asphalt used in EME13, SBS-modified asphalt can simultaneously increase the failure strain and failure strength of the asphalt mixture, enabling Sup13 to exhibit the highest SED.

#### 4.2.4. IDEAL-CT Test Results

The IDEAL-CT test results are shown in Table 6, with each result being the average of four parallel specimens. Here, G_f_ represents the fracture energy consumed from initial loading to complete failure; ∣m75∣ is the slope of the force-displacement curve in the latter half of loading, which can indirectly reflect the crack propagation rate; and CT_index_ can be regarded as a comprehensive indicator of the specimen’s cracking resistance.

As shown in Table 6, (1) with the increase in RAP content in EME13, its cracking resistance shows a reasonable regularity, that is, the G_f_ and CT_index_ initially decrease and then increase, while ∣m75∣ initially increases and then decreases, with the turning point appearing at the RAP content of 40%. This behavior arises from two competing mechanisms: First, weak interfacial bonding between RAP and virgin asphalt creates stress concentration zones, which hurt the cracking resistance of EME13 [27,28]. This is the reason for the continuous decline in the cracking resistance of EME13 when the RAP content does not exceed 40%. Second, from the asphalt binder test results, it is known that the performance of reclaimed asphalt is quite close to that of hard asphalt, and even some properties of reclaimed asphalt are slightly better than those of hard asphalt due to the presence of partially undegraded SBS modifiers. This fact means that the addition of RAP may positively impact the cracking resistance of EME13, which is why the cracking resistance of EME-60 is slightly better than that of EME-40. Therefore, it can be said that the impact of RAP on the cracking resistance of EME13 is the result of the combined effects of the above two aspects, which also leads to the parabolic changes in the cracking resistance-related parameters in Table 6. In addition, it can be found that although the RAP content impacts the cracking resistance of EME13, whether it is an increase or a decrease, the impact is within 30%. (2) Compared with EME13, Sup13 shows better cracking resistance, which is related to using SBS-modified asphalt in Sup13. However, the finer grading and higher asphalt content of EME13 are conducive to improving its cracking resistance, making the CT_index_ of Sup13 only 15.9% higher than that of EME-0.

#### 4.2.5. Dynamic Modulus Test Results

**Figure 8:**
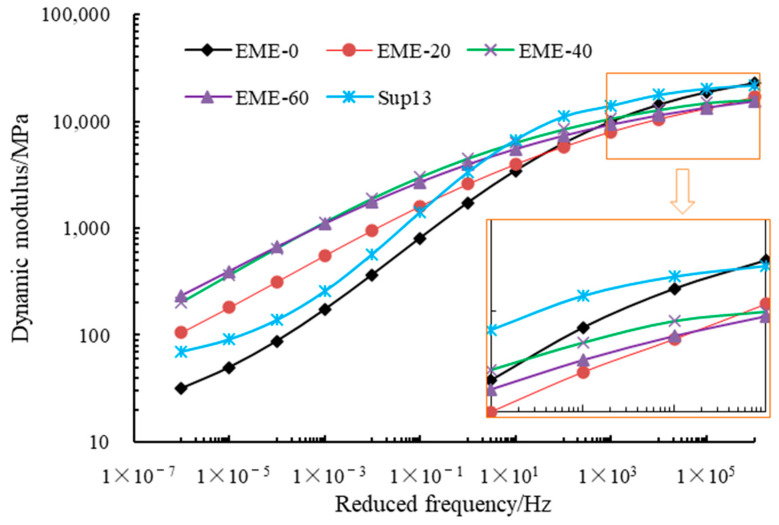
The dynamic modulus master curves of different asphalt mixtures.

To facilitate a comparative analysis of the dynamic modulus of different asphalt mixtures, this study employed the Mastersolver tool (developed under the NCHRP 9-29 project) to fit the dynamic modulus master curves. Mastersolver utilizes a sigmoidal function as the objective function to fit dynamic modulus test results across varying temperatures and frequencies. The reference temperature for fitting was set to 20 °C. The fitting results are presented in Figure 8.

As shown in Figure 8, (1) the dynamic modulus of EME-13 is positively correlated with the RAP content at the low-frequency region. For example, at 10^−6^ Hz, when the RAP content increases from 20% to 60%, the dynamic modulus of EME-13 increases by 237%, 536%, and 641%, respectively. At the high-frequency region, the dynamic modulus of EME-13 is negatively correlated with the RAP content. For example, at 10^6^ Hz, when the RAP content increases from 20% to 60%, the dynamic modulus of EME-13 decreases by 25.6%, 29.5%, and 31.9%, respectively. According to the time-temperature superposition principle of polymers, the dynamic modulus at the low-frequency region is equivalent to that at high temperatures, while the dynamic modulus at the high-frequency region is equivalent to that at low temperatures. Therefore, RAP increases the modulus of EME-13 at high temperatures while decreasing it at low temperatures, which may be related to the different rheological properties of reclaimed and hard asphalt. For high-modulus asphalt mixtures, the modulus is represented by the modulus at 10 Hz. As shown in Figure 8, at 10 Hz, the dynamic modulus of EME-13 increases with RAP content. When the RAP content increases from 20% to 60%, the dynamic modulus of EME-13 increases by 15.1%, 59.8%, and 83.6%, respectively. This indicates that using RAP makes it easier for asphalt mixtures to meet the modulus requirements of high-modulus asphalt mixtures, which is beneficial for the recycling of RAP in such applications. (2) Compared with EME-13, the dynamic modulus of Sup-13 is lower than the former at the low-frequency region (except for EME-0) but higher at the high-frequency region. This is related to the use of modified asphalt in Sup-13, which fundamentally alters its rheological behavior compared to pure asphalt.

Dynamic modulus is closely related to the pavement performance of asphalt mixtures. Generally speaking, the higher the dynamic modulus, the better the rutting resistance of asphalt mixtures. Some studies suggest that dynamic modulus is negatively correlated with the fatigue life of asphalt mixtures. Under the same strain level, asphalt mixtures with higher dynamic modulus bear higher stress, thus generating greater distress and having lower fatigue life [29]. However, other researchers hold the opposite view. They believe that under the same traffic load, asphalt mixtures with higher dynamic modulus produce smaller strains, and therefore, they suffer less distress and have higher fatigue life [30]. Thus, changes in dynamic modulus do not necessarily lead to an increase or decrease in fatigue life, which is part of why some researchers have begun to explore other indicators to characterize the fatigue performance of asphalt mixtures [31].

#### 4.2.6. Comprehensive Analysis

To comprehensively compare the characteristics of the various asphalt mixtures, this paper calculated their material costs, with the results presented in Table 7. The unit price of hard asphalt is 500 USD/ton, the unit price of SBS asphalt is 710 USD/ton, and the unit price of aggregate is 30 USD/ton. The price information was obtained by consulting pavement contractors in Yangzhou, Jiangsu Province, and represents approximately the average prices over the past year. The asphalt content is shown in Table 5.

As shown in Table 7, EME-13 is significantly more cost-effective than Sup-13. This is primarily due to its use of lower-cost hard asphalt compared to the more expensive SBS-modified asphalt in Sup13. Moreover, the higher the RAP content, the more pronounced the cost-effectiveness becomes. When the RAP content reaches 60%, the cost is even less than half of that of Sup-13. It should be noted that using RAP in asphalt mixtures involves more than just material costs. There are many hidden costs. For example, (1) processing and transporting RAP add expenses; (2) performance may vary due to RAP inconsistency; (3) mixing plant operations may need adjustments, increasing production costs; and (4) government environmental policies could further impact economics. All these affect the economy of RAP asphalt mixtures. Due to our research limitations, these costs are not included in the economic analysis. Even accounting for these additional costs, the financial benefits of these mixtures remain substantial.

**Figure 9:**
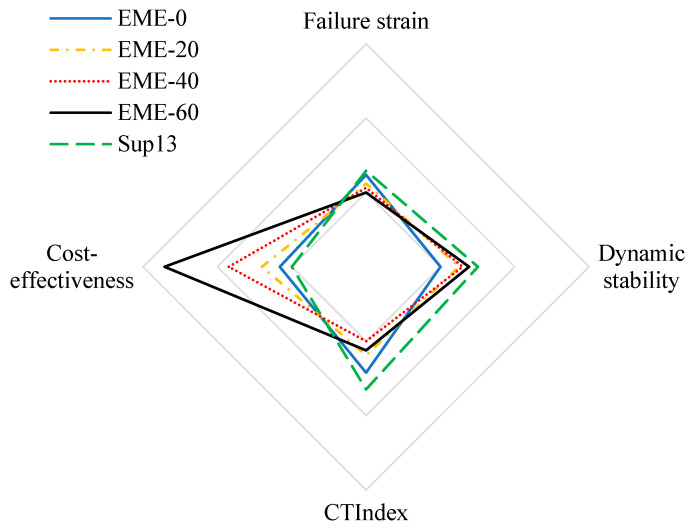
Comparison of indicators for different asphalt mixtures.

This paper further drew the radar charts to evaluate key performance indicators across different asphalt mixtures, including rutting resistance, low-temperature cracking resistance, medium-temperature cracking resistance, and cost-effectiveness. The results are shown in Figure 9, with all data normalized for standardized visualization.

As can be seen from Figure 9, while Sup13 demonstrates optimal pavement performance, it also carries the highest cost. With the increase in RAP content, the price of EME-13 drops significantly, its high-temperature performance improves, and its cracking resistance decreases slightly, showing a favorable cost-performance balance.

## 5. Conclusions

This study investigated the feasibility of high-content RAP utilization using the High-modulus Asphalt Mixture design methodology. The key findings are as follows:

(1) The technical performance and chemical functional groups of reclaimed asphalt are similar to those of hard asphalt, indicating that it is feasible to replace hard asphalt with reclaimed asphalt in the design of High-modulus Asphalt Mixtures.
(2) With the increase in RAP content, the high-temperature performance of EME-13 increases by 20% to 60%; the low-temperature and medium-temperature cracking resistance decreases slightly by 10% to 20%; the dynamic modulus in the low-frequency range increases by 3 to 6 times, while the dynamic modulus in the high-frequency range decreases by 20% to 30%. RAP makes it easier for EME-13 to meet the modulus design requirements of High-modulus Asphalt Mixtures.
(3) The pavement performance of modified asphalt Sup-13 is superior to that of EME-13, but its cost is also the highest. EME-13 with high RAP content has a significant economic advantage while having slightly lower pavement performance than Sup-13, making it highly cost-effective.
(4) The aged characteristics of RAP binder are strategically compatible with the design requirements of High-modulus Asphalt Mixtures, which confirms the technical and economic viability of incorporating a high content (up to 60%) of RAP.

## Figures and Tables

**Table 1:** Sieve analysis of aggregates.

Aggregate Size (mm)	Percentage Passing (%) at the Following Sieves (mm)									
	16.0	13.2	9.5	4.75	2.36	1.18	0.6	0.3	0.15	0.075
10–15	100	86.2	30.7	4.6	0.5	0.1	0.1	0.1	0.1	0.1
5–10	100	94.9	76.8	5.1	0.6	0.5	0.3	0.3	0.3	0.3
3–5	100	100	100	58.1	12.9	7.8	6.4	4.8	1.6	0.8
0–3	100	100	100	95.1	72.4	51.9	31.8	15.7	11.5	8.1
RAP	100	98	90.2	67.9	47	35.5	22.4	13.1	9	3.4
Filler	100	100	100	100	100	100	100	100	96.5	83.6

**Table 2:** Basic properties of different asphalt.

Asphalt Type	Basic Properties		
	Penetration (0.1 mm)	Softening Point (°C)	Ductility (cm)
SBS asphalt	56.3	81.5	30.4
Reclaimed asphalt	28.6	67.0	3.2
Hard asphalt	25.7	63.5	4.5

**Table 3:** LAS results of different asphalt.

Type of Asphalt	Modal A	Modal B	Nf at Different Strains (ε)		
			2.50	5.00	10.00
SBS asphalt	1,380,000	2.784	107,652	15,626	2268
Reclaimed asphalt	1,082,300	3.411	47,531	4468	420
Hard asphalt	739,700	3.463	30,969	2808	255

**Table 4:** Gradation of different asphalt mixtures.

Mixture Type	Percentage Passing (%) at the Following Sieves (mm)									
	16.0	13.2	9.5	4.75	2.36	1.18	0.6	0.3	0.15	0.075
EME-0	100.0	95.8	77.6	46.0	29.1	22.3	16.0	11.6	9.3	6.3
EME-20	100.0	95.5	77.7	47.5	29.8	22.9	16.4	11.3	9.0	6.5
EME-40	100.0	95.6	78.5	46.4	28.8	22.5	16.3	11.7	9.2	6.1
EME-60	100.0	95.2	76.5	46.5	29.4	23.4	17.5	11.9	9.3	5.9
Sup13	100	94.3	69.2	44.3	34.1	24.1	15.8	8.9	7.0	5.1

**Table 5:** Asphalt mixture design results.

Mixture Type	RAP Content/%	Asphalt Content (%)		Air Void (%)/
		Recycled Asphalt Content (%)	Extra Asphalt Content (%)	
EME-0	0	0	5.3	3.2
EME-20	20	1.0	4.3	3.3
EME-40	40	2.0	3.3	3.2
EME-60	60	3.0	2.3	3.1
Sup13	0	0	4.9	4.0

**Table 6:** IDEAL-CT test results of different asphalt mixtures.

Mixture Type	G_f_ (J/m^2^)	|m_75_|	CT_Index_
EME-0	14,380.3	1.90	257.3
EME-20	10,983.2	1.95	214.8
EME-40	7982.5	2.11	180.9
EME-60	8313.4	2.01	203.1
Sup13	11,372.3	1.67	298.2

**Table 7:** Price of different asphalt mixtures.

Mixture Type	EME-0	EME-20	EME-40	EME-60	Sup13
Price (USD/ton)	54.9	45.5	34.5	23.5	63.3

## Data Availability

The original contributions presented in this study are included in the article. Further inquiries can be directed to the corresponding author.
